# Chronic Specific Urethritis*Read at the meeting of the Alumni Association, Niagara University, May 12, 1897; Physicians’ Club, March 24, 1897.

**Published:** 1897-09

**Authors:** J. Henry Dowd


### Selections and Abstracts

### CHRONIC SPECIFIC URETHRITIS. *

Heretofore I have refrained from writing on the subject of chronic specific urethritis, because it is a practice that necessitates the possession of a lot of expensive instruments, which except in the hands of a person using the same several times daily, would become a white elephant. The care of the same, together with the modus operandi, demands time which the general practitioner can not spare, and unless the technique is carefully followed you can not hope to have the result wished —namely, a cure.

The male urethra has an average length of 8½ inches to 9 inches, and for convenience I will divide it into two portions,—anterior and posterior; the anterior being that portion from the meatus to the membranous, and is about 5 to 6 inches in length. Here we find a quite powerful voluntary muscle, the compressor urethræ, this being about ½ to ¾ inch in length, surrounding the membranous portion, and dividing the canal into two distinct parts. Behind this and about two and a half inches long, the urethra is continued through the prostate to to the vesicle opening, and this is called the posterior urethra.

The pathology of chronic urethritis I will pass by with a few words. The most important pathological condition found is localized foci of inflammation, extending in depth according to the age of the patient This is usually accompanied by congestion of the whole mucous tract. From these local foci sometimes spring granulations, ulceration may result, and lastly involvement of one or several follicles, or the prostatic sinuses may act as a fortress from which the enemy may pour out shot in the form of a morning drop, with little or no chance of destruction, except by instruments I will later describe, which must penetrate to the deepest recesses.

It might be well here to review briefly the anatomy from within out:—epithelium, which consists of three layers; subepithelial tissue, consisting of connective tissue; blood and lymph vessels; mucous membrane; sub-mucous tissue; muscular, two layers, and cavernous tissue. The mucous membrane is studded with small cavities, called follicles; in these at times may lurk the germs which make a urethritis indefinite. Half inch from the meatus is a small band-like projection,

* Read at the meeting of the Alumni Association, Niagara University, May 12, 1897; Physicians' Club, March 24, 1897.

sometimes well developed. Do not diagnosticate it as a stricture—it is the navicular valve. Nothing now is found of importance until the posterior urethra is reached, where we find the openings of the ejaculatory ducts, prostatic sinuses, caput gallinaginis and innumerable follicles. The mucous membrane varies in color, being from the meatus backward as follows: whitish, light pink, pink, until at the compressor muscle it is a very bright red. The posterior urethra is also very red, almost resembling blood.

The symptoms of chronic urethritis I consider unnecessary to repeat. Let me answer a question I am frequently asked, and it will suffice for the objective symptoms. When is a man cured of gonorrhea? When his morning urine is in the same condition as before he had the first attack, viz.:—perfectly clean and free from any sediment. Exceptions—if there should be one almost colorless floating shred, and the microscope shows it to consist of only mucus and epithelial cells. For as long as these shreds contain pus or gonococcus, the man is a dangerous individual. The diagnosis is comparatively easy, for from the history one should at once know whether he is to treat a chronic urethritis or a urethral chancre. The diagnosis of the portion of the urethra affected is quite a valuable aid in treatment.

As I have already said, the compressor muscle divides the canal into two parts. Any secretions formed anterior can not gravitate beyond this muscle, and any behind can not come forward. It is here our two-beaker test plays an important part. Have the patient urinate in two glasses. The first is turbid, and contains small shred-like pieces. The second portion is also turbid, but less so than the first. This shows an antero-posterior involvement. Should the second contain small comma-like plugs, it indicates the prostatic sinuses are involved, and as the bladder contracts to expel the last few drops of urine, these were squeezed out.

Now, supposing the first glass to be turbid and containing shreds, but the second clear. Generally speaking, this shows anterior trouble only; but not always, for if the posterior urethra is not involved sufficiently to cause adequate destruction of epithelium and the like, so it can readily flow backward and mix with the urine in the bladder, it will be washed out by the first stream, thus rendering the second clear, and deceiving us in our diagnosis. Taking into consideration the history of the case and the amount of turbidity of the first specimen, I always on general principles medicate the posterior urethra; for I think I am safe in saying that 90 per cent. of all cases become posterior when they are four weeks or over in age. If you want to be very positive, a catheter can be passed as far as the compressor muscle, and the anterior urethra washed out with clear water. Or with a small syringe inject the anterior urethra with a methyl blue, and hold for a few minutes. Thus the shreds and pus will be colored, and if any are not, you may be sure they come from behind the cut-off muscle.

We have now, as you have seen, ascertained the cause, pathological condition, and region or regions affected. How shall we cure is the question. It is an undisputed fact that after a certain time the gonococcus becomes dormant—moribund, so to speak; and especially is this marked when their field of cultivation is rendered uncultivatable. But let this field be fertilized by irritation or an excess of blood to the part, and they, as a person feeble from age, who by a stimulant is revived, also revive for a time, but very soon relapse into their former condition. Now, *vice versa* as with a person, if we render their field uninhabitable, remove all stimulation, and cause their lives to be unhappy, they must do one thing—namely, die. And they will die in quickness, according to the age of the case and depth to which they have penetrated. How can this be done? Not by injections of solutions which will keep up an irritation, medicines which will cause depletion for the time being, or by allowing the patient to use liquors or enjoy amusements which will cause congestion of the parts.

We have a very sensitive, long-continued, irritated mucous membrane, which to be brought to a normal condition must be treated as a refractory baby, that is, humored. For in this way only can we hope for any result.

You will notice the solutions which I use are many, many times weaker than those often prescribed for the same condition. But if they are cautiously used in gradually increasing strength, there will be but one outcome,—urine cleaning, shreds diminishing from day to day, discharge stopped, case cured. I shall endeavor to make this so plain and untechnical that the average practitioner will be able to treat successfully a large proportion of the cases that he meets in his daily or yearly practice. The armamentarium necessary is a microscope with a 1-12 oil em. objective and accessories; an Ultzman syringe, capacity 6 oz.; semi-soft catheter, No. 15 F., with eye near end, a shield for same; set of steel sounds, numbering from 20 to 30 F.; a sterilizer and glycerine. Never use any oily substance to lubricate the catheter or sounds, as it will so coat the mucous membrane as to prevent the solution from coming in contact with the same. Now, if you want to be prepared to treat all cases which you will see, an electric light, endoscopic tubes, swabs, applicators, probes, long hypodermic needle and polypus forceps are absolutely necessary.

Let me treat a case for you, on paper. The manner is simply modification of the Ultzman with additions. History, six to eight weeks old; constant discharge, especially marked in the morning, or possibly only a morning drop. If you are going to apply the test already mentioned for posterior urethritis, do not allow the patient to pass urine. If not, have him pass it in two glasses. The first contains shreds and mucus, also the second, but not mucous plugs. It is not an absolute necessity at this time to examine for gonococcus, but it is good policy so to do. The first solution I call the zinc alu. carb., which consists of zinc sulphate, alum and acid carbolic, of each ½ dram in 6 oz., 2 drams distilled water, this being simply a modified formula of Ultzman's solution. Of this solution use ½ oz to 7½ oz. distilled water for first irrigation. After having the canal perfectly clean by previous urination, a sterilized catheter should be passed as far as the cut-off muscle. Attach the syringe and expel air from catheter, after which push it through the cut-off, so the eye will rest just inside the same and immediately at beginning of the prostatic or posterior urethra. Gently push the piston forward, depositing about 3 oz. of the solution on the prostatic mucous membrane. This will readily flow backward into the bladder. Now withdraw catheter to anterior border of cut-off muscle, having patient hold meatus at intervals so as to distend canal, and bring the solution in contact with all folds. Deposit the remainder in the anterior urethra. When the patient empties his bladder the solution which you have previously left therein will, of course, flow out over the mucous membrane, thus again medicating it. On the second day use the same solution, 6 drams to distilled water, 7½ oz. Third day, 12 drams to 7½ ounces Fourth day, 12 drams to 4½ oz. Now this is as strong as this solution should be used.

If it has been used cautiously, adhering strictly to the above remarks as to passage of catheter and antisepsis, the case, if recent, should show marked improvement. The discharge should be nearly if not wholly stopped, and nothing remain but a slightly turbid urine with a few fine shreds floating therein. There is but one thing that can remove these shreds —pressure; but pressure must not be used until we have a perfectly clean urine, containing only shreds. Therefore, the mucous membrane must still receive attention.

At this stage the best, in my opinion, the only remedy for congestion or inflammation of the urinary membrane, is silver nitrate; but it must be used very weak until the surface over which it flows is almost normal, this being evident by the condition of the fluid which washes it.

With the same procedure as with the former solution, the first day I use a 1 to 12,000 solution; the second day, 1 to 10,000; the third day, 1 to 8,000; the fourth day, 1 to 6,000. Now by this time the urine is generally perfectly clear of any substance except the shreds, and you must resort to some measure to remove them. Shreds, in their early stage, generally speaking, consist of exfoliated epithelium, pus, mucus, gonococci and other cocci peculiar to pus-producing localities. They are due to an underlying inflammation which is constantly undergoing changes, throwing off the dead or dying upper layer, which is the epithelium; and as I have said before, this localized inflammation can only be removed by pressure. If the case is a very old one it is well to use a urethrometer and search for stricture. Should none be found, the largest sound that will comfortably pass the meatus should be sterilized, lubricated with glycerine, and passed into the bladder, allowing it to remain for five minutes, after which remove, and with a catheter in same way as previously, use silver solution 1 to 5,000. To pass the sound oftener than every four or five days would cause too much irritation; but on the second day you can irrigate again, using solution 1 to 4,000.

The urine should receive close attention as to clearness, and I am sure when you have the canal dilated to at least thirty, followed each time by an irrigation of silver, which should be gradually increased in strength from the last mentioned up to 1 to 1,000 or 1 to 800, you will find a urine containing only one or two very light-colored shreds, which will float, this indicating that they are composed chiefly of mucus and epithelium.

It is now the microscope should come into play, and these shreds thoroughly examined for gonococcus and pus, and if found, the dilation and irrigation should be continued. Or, as I shall later describe, the electro-endoscope may come in as a valuable adjunct.

Print these words on your brain in capital letters—as long as a shred contains gonococci and pus cells, that patient is a dangerous individual—dangerous not only to himself, but to every female, let it be wife or mistress, with whom he comes in contact sexually. On the other hand, if these shreds consist of mucus and epithelium, it shows a local spot of plastic infiltration, which is not a source of infection, but may contract and cause stricture in time to come.

Now, supposing that after six or eight treatments the urine has been clean both of mucus and shreds for, say three days, there has been no discharge except one drop in the morning, this being pure pus and when examined is found to contain gonococci. For this condition there is usually but one cause, follicular involvement, where the gonococci are still living in luxury; but so as not to overpopulate a fertile country, emigration takes place once in twenty-four hours. To cure this condition quickly there is but one remedy—find the enemy in his den and completely annihilate him. This can only be accomplished by means of the electric light and a tube which will enable you to find these follicles and destroy their inhabitants with a 10 to 15 per cent. solution of silver. Should one or several large shreds containing pus and epithelium persist after repeated use of full-sized sounds, it is very probable that you may find what is called granular spots. These also should be destroyed by local applications of silver.

GENERAL TREATMENT.

Should there be a pin hole or even small meatus, it should be divided up to 29 or 30 F. Strictures should be dilated after the inflammation around is subdued, this being evident by the clearness of the urine. With a profuse discharge, a weak solution of copper or zinc may be of benefit, but in my opinion is of very little. Injections do more harm than good, and I never use them. Medicine internally is unnecessary, excepting in a debilitated patient. Iron or cod liver oil may have a pleasing effect in bracing up a lowered vitality. Syphilitics should always receive constitutional treatment, pushing it to its utmost. When the urine is excessively acid and examination shows either oxalate of lime or uric acid, a well-regulated diet for these condition, with some anti-lithemics, paying especial attenion to the intestinal canal and digestive system, is of all importance.

CONCLUSIONS.

First, 99 per cent. of patients can be cured. Second, examine the urine visually every time you see the patient. Third, soothe his delicate mucous membrane by very weak solutions, rather than irritate it by injections which, if used, certainly do. Fourth, never use an instrument except catheter, until the urine contains only shreds floating in a clear liquid. Fifth, never discharge a case as cured until the urine is the same as before he had the trouble, viz.: clean. Or if one or two small shreds, examine carefully to see they contain no pus or gonococci. You may save some woman's tubes or ovaries, and unspeakable suffering. Sixth, don't use the electric light to impress the patient with your greatness. If not called for, it is a positive damage. Seventh, silver nitrate will not cure every case, but it will cure 96 per cent. and is the remedy *par excellence*. Eighth, adhere to cleanliness, and by this I mean sterilization, for germs are the cause of inflammation, and the introduction of unclean catheters, sounds, and the like, will cause the same.—J. HENRY DOWD, M. D., in *Buffalo Medical Journal*.

